# Computational methods for detecting copy number variations in cancer genome using next generation sequencing: principles and challenges

**DOI:** 10.18632/oncotarget.1537

**Published:** 2013-11-16

**Authors:** Biao Liu, Carl D. Morrison, Candace S. Johnson, Donald L. Trump, Maochun Qin, Jeffrey C. Conroy, Jianmin Wang, Song Liu

**Affiliations:** ^1^ Center for Personalized Medicine, Roswell Park Cancer Institute, Buffalo, NY; ^2^ Department of Biostatistics and Bioinformatics, Roswell Park Cancer Institute, Buffalo, NY; ^3^ Department of Pathology, Roswell Park Cancer Institute, Buffalo, NY; ^4^ Department of Pharmacology and Therapeutics, Roswell Park Cancer Institute, Buffalo, NY; ^5^ Department of Medicine, Roswell Park Cancer Institute, Buffalo, NY; ^6^ Department of Cancer Genetics, Roswell Park Cancer Institute, Buffalo, NY

**Keywords:** copy number variation, next generation sequencing, cancer genome analysis, somatic mutations

## Abstract

Accurate detection of somatic copy number variations (CNVs) is an essential part of cancer genome analysis, and plays an important role in oncotarget identifications. Next generation sequencing (NGS) holds the promise to revolutionize somatic CNV detection. In this review, we provide an overview of current analytic tools used for CNV detection in NGS-based cancer studies. We summarize the NGS data types used for CNV detection, decipher the principles for data preprocessing, segmentation, and interpretation, and discuss the challenges in somatic CNV detection. This review aims to provide a guide to the analytic tools used in NGS-based cancer CNV studies, and to discuss the important factors that researchers need to consider when analyzing NGS data for somatic CNV detections.

Tumors usually rise from normal cells with tissue specific acquired mutations or aberrations in their genomic materials [1]. Copy number variation (CNV) is one of the most important somatic aberrations [1-4]. CNV was initially defined as the amplification or deletion of genetic materials in the size of >1kb [5, 6], then was widened to include much smaller events (>50 bp) [7] on accounting of the greatly improved resolution of detection methods. Tumor genomes usually acquire somatic CNVs during carcinogenesis, and the amplification of oncogenes or deletion of tumor suppressor genes are usually pathogenic, as the expression level of a gene is highly correlated to its copy number [8]. In searching for oncotargets, genomic regions with recurrent CNVs in tumor genomes are believed to have high probability of containing cancer genes [9]. Indeed, quite a few cancer-related genes have been identified to be affected by somatic CNVs [4, 10-13]. This list of CNV related oncotargets includes *ERBB2, EGFR, MYC, PIK3CA, IGF1R, FGFR1/2, KRAS, CDK4, CCND1, MDM2, MET, CDK6* for amplification, and *RB1, PTEN, CDKN2A/B, ARID1A, MAP2K4, NF1, SMAD4, BRCA1/2, MSH2/6, DCC, CDH1* for deletion. Different patterns of somatic mutations may divide one type of cancer into different subgroups, and the prognostics and treatment responses of the subgroups could be very different [8, 14]. For example, Trastuzumab is effective only to breast cancers in which *ERBB2* is amplified and over expressed [15-18]. The identification of such somatic events should facilitate prognosis and treatment decision [14]. Therefore, accurate CNVs detection is an essential part of cancer genome analysis, which holds great promise to improve cancer diagnosis and treatment decision.

An ideal CNV detection method should accurately quantify the copy numbers in all genomic segments and delineate their breakpoints across the whole genome. Currently, several platforms with different achievable throughputs, coverage, and resolutions are available, including Fluorescence *In Situ* Hybridization (FISH) [19, 20], NanoString's digital detection technology [21-23], array comparative genomic hybridization (array CGH) [24], Single Nucleotide Polymorphism (SNP) array [7], and Next Generation Sequencing (NGS) [25-33]. In this Review, we focus on NGS-based approaches, as they have been emerging as the primary means of interrogating the CNV in recent investigations. Somatic CNV detection in cancer is our primary focus, as the characteristics of somatic CNVs need special consideration in algorithms and strategies in which germline CNV detection programs are usually not suited for. We begin by briefly reviewing the NGS studies and outlining the existing computational programs for somatic CNV detection. We then cover the primary types of NGS data that could be used in CNV detection, followed by deciphering and summarizing the principles under data preprocessing, segmentation, and interpretation. The key similarity and difference between different computational programs are described. We continue by providing some discussion of the challenges in somatic CNV detection, and we conclude with an outlook on the near future of this fast evolving field. The aims of this article are to provide a guide to the analytic tools used in NGS-based cancer CNV studies, and to discuss the important factors that researchers need to consider when analyzing NGS data for somatic CNV detections.

## NGS Studies

NGS is a technology that parallelly sequences massive amounts of short DNA strands from randomly fragmented copies of a genome [25-33]. A typical NGS run will generate millions to billions of reads, which are assumed to be random representations of the targeted regions or the whole genome. The widespread availability of NGS technology provides an unprecedented opportunity to systematically screen for CNVs. NGS is flexible in that it can be adapted to cover either the whole genome or targeted regions of interest (for example, the exome, defined as the complete set of coding regions of human genome). NGS-based CNV studies frequently fall into Whole Genome Sequencing (WGS) studies and Whole Exome Sequencing (WES) studies, and they are both considered in this Review.

### WGS

A single experiment of WGS can produce multidimensional information for discovering CNVs in a genome-wide scale. First, as the frequency of a genomic region being represented by reads is linear correlated to its copy number in a broad range after correcting some systematic bias (such as GC bias and mappability bias), the analysis of relative number of reads falling in a region can reveal its copy number. Second, the numbers of reads covering both alleles at a single nucleotide polymorphism (SNP) locus could be used to estimate the allele specific absolute copy numbers, determine copy neutral regions of loss of heterozygosity (LOH), and infer the amount of normal cells in a tumor population. Third, reads that capture the sequences of boundary regions can serve as signatures of structural variations, and identify the breakpoints of structure variations at base pair resolution. Fourth, as pair-end and mate-paired sequencing are often employed, the pairs of reads with spans and/or orientations are inconsistent with the reference genome can facilitate the determination of structure variations. A more detailed discussion about these data types is followed. The quality and richness of data make WGS by far the most powerful approach for CNV detection.

### WES

The cost of a WGS experiment has dropped substantially in the past several years, but it is still relatively expensive (>$5,000 per sample), and the resultant data requires substantial investment in computational resources for processing and storage. To balance the cost and output, WES approach can be used when WGS is not financially affordable. The exome represents a highly function-enriched subset of the human genome, and CNVs in exome are more likely to be pathogenic than those in nongenic regions [34, 35]. In WES experiments, DNA fragments belonging to the exome can be enriched from fragmented genome sample by hybridization with designed probes or by PCR amplification with designed primers, and then parallel sequencing is applied to the selected fragments. Compared with WGS, WES cannot reach base pair resolution in determining breakpoints falling into non-coding regions due to the discrete nature of exome regions, and its CNV calling results are only reliable in exon-rich regions because of the uneven distribution of exons across the genome. Nevertheless, WES is ideal for searching for gene-harboring CNVs in a cost-efficient and analytic-effective manner. As the data types from WES are similar to WGS, similar data processing is usually conducted.

## Somatic CNV Detection Programs for NGS data

Sophisticated computational algorithms are crucial to accurately retrieve segmental copy number and breaking points from NGS data. Although the NGS technology was only emerging and applied to cancer studies during the past several years [36-56], a number of somatic CNV detection programs for NGS data have been developed. In table 1, we list 11 publicly available programs and their websites. SegSeq [41], ReadDepth [57], BICseq [58], Patchwalk [59], OncoSNP-SEQ [60], HMMCOPY, and CONSERTING were designed for WGS data; ExomeCNV [61], VarScan2 [62], and HAPSEG/ABSOLUTE [63, 64] can be applied to WES data. Control-FREEC [65, 66] can analyze the data from both WES and WGS platforms.

**Table 1:** Available programs for detecting copy number variation in cancer genome using next generation sequencing data

Platform	Program	Website	Ref.	Year	Language
WGS	SegSeq	http://www.broadinstitute.org/cancer/cga/Home	[41]	2009	MATLAB
	ReadDepth	http://code.google.com/p/readdepth/	[57]	2011	R
	BIC-seq	http://compbio.med.harvard.edu/Supplements/PNAS11.html	[58]	2011	Perl/R
	Patchwork	http://patchwork.r-forge.r-project.org/	[59]	2013	R
	OncoSNP-SEQ	https://sites.google.com/site/oncosnpseq/	[60]	2013	MATLAB
	HMMcopy	http://compbio.bccrc.ca/software/hmmcopy/	/	/	R
	CONSERTING	http://www.stjuderesearch.org/site/lab/zhang	/	/	R
WES	ExomeCNV	https://secure.genome.ucla.edu/index.php/ExomeCNV_User_Guide	[61]	2011	R
	VarScan2	http://varscan.sourceforge.net/	[62]	2012	Java
	HAPSEG/ABSOLUTE	http://www.broadinstitute.org/cancer/cga/Home	[63, 64]	2012	R
WGS&WES	Control_FREEC	http://bioinfo-out.curie.fr/projects/freec/#documentation	[65, 66]	2011	C

**Figure 1:**
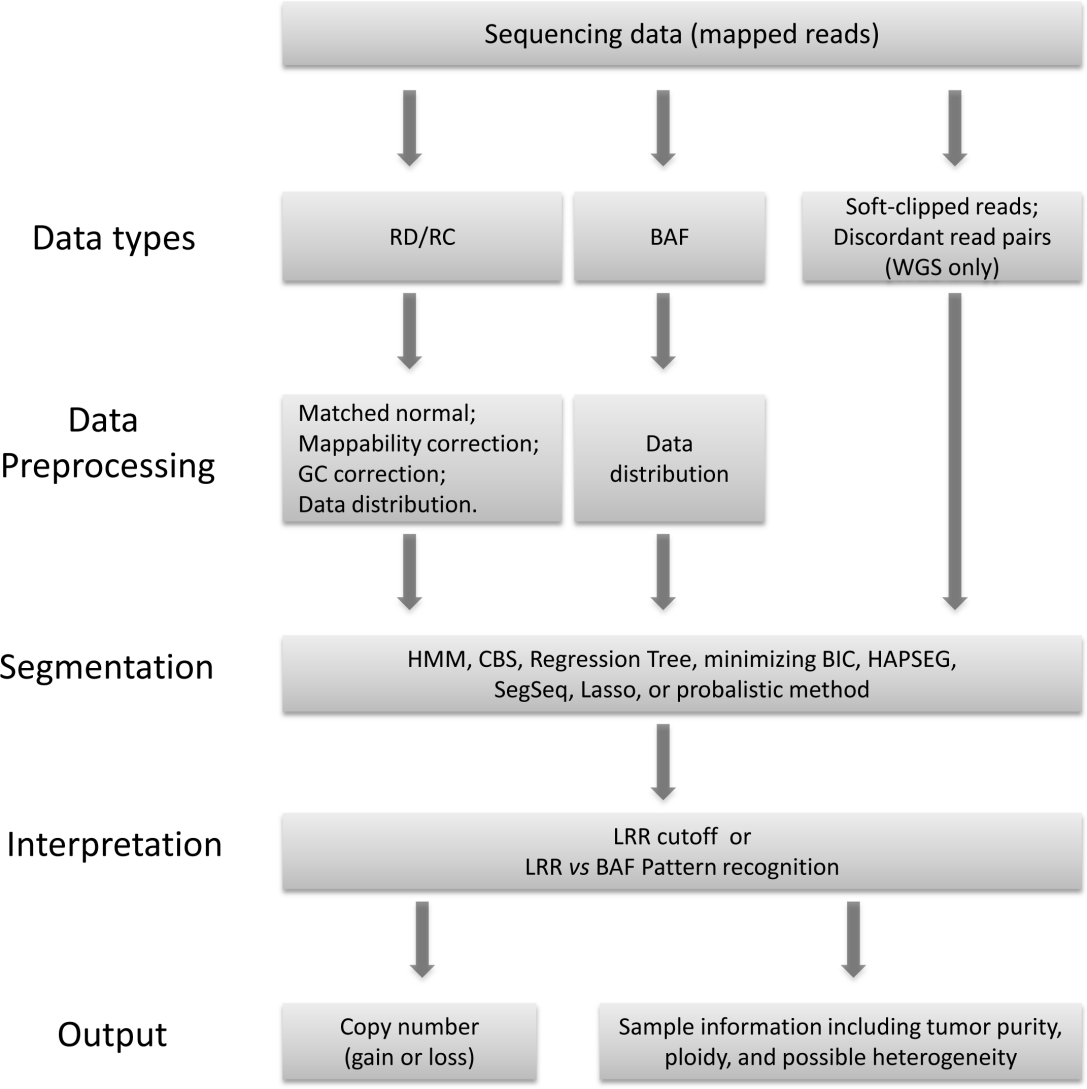
The workflow chart that computational methods fall in for calling somatic copy number variations from next generation sequencing data

A somatic CNV detection program generally follows the flow chart illustrated in Figure 1. It may take one or more data types as inputs. The core of a computational algorithm can be broadly divided into three modules: data preprocessing, segmentation, and interpretation. Different strategies could be used in each module. Depending on the embedded algorithms, the programs output the results at different level of details. Some programs only report the segments with copy number gain or loss, some report segments with total or allele specific copy number, and others provide further information including tumor purity, ploidy, and even heterogeneity. The major features of each program have been summarized in Table 2, and we will discuss them with more details in the following.

**Table 2:** Major features of programs for detecting copy number variation in cancer genome using next generation sequencing data^1^

Programs	Data type	Data preprocessing^3^	Segmentation	Interpretation	Sample information
SegSeq	RC	Matched normal;	Local change-point analysis with a subsequent merging procedure	Optimized cutoffs	/
ReadDepth	RD Discordant read pairs	Mappability correction; GC correction; RD Negative-binomial distribution	CBS	Optimized cutoffs	/
BIC-seq	RD	Matched normal; No data distribution assumption	Minimizing BIC	Empirical cutoffs	/
Patchwork	RD BAF	Normal genome; GC correction	CBS	Pattern Recognition and empirical cutoffs	Tumor purity Tumor ploidy
OncoSNP-SEQ	RC BAF	Matched normal; Mappability correction; GC correction Mixture of uniform and binomial distribution	HMM	HMM	Tumor purity Tumor ploidy Tumor heterogeneity
HMMcopy	RC	Matched normal; Mappability correction; GC correction	HMM	HMM	/
CONSERTING	RD BAF Soft-clipped reads	Matched normal; Mappability filtering; GC correction	Regression Tree	Empirical cutoff	/
ExomeCNV	RD^2^	Matched normal	CBS	Optimized cutoff	Fixed tumor purity
VarScan^2^	RD	Matched normal	CBS	Empirical cutoff	/
HAPSEG/ABSOLUTE	RD at SNP loci	Matched normal	probabilistic method	Pattern Matching and fit platform error model	Tumor purity Tumor ploidy Existence of sub-clone
Control_FREEC	RC	Matched normal and/or GC and Mappability correction;	LASSO algorithm	Empirical cutoff	Tumor purity User inputs tumor ploidy

1 Abbreviations: RC, Read Counts; RD, Read Depth; BAF, B Allele Frequency; SNP, single nucleotide polymorphism; CBS, circular binary segmentation; HMM, hidden Markov model.

2 ExomeCNV uses only RD for calling CNV; it uses BAF for calling LOH.

3 The data is assumed to be in normal distribution if not specified.

## Data Types

In this section, we describe the types of data that could be used in NGS-based CNV detection. These data types include read counts (RC) or read depth (RD), B Allele Frequency (BAF), soft-clipped reads, and discordant read pairs.

### RD or RC

**Figure 2:**
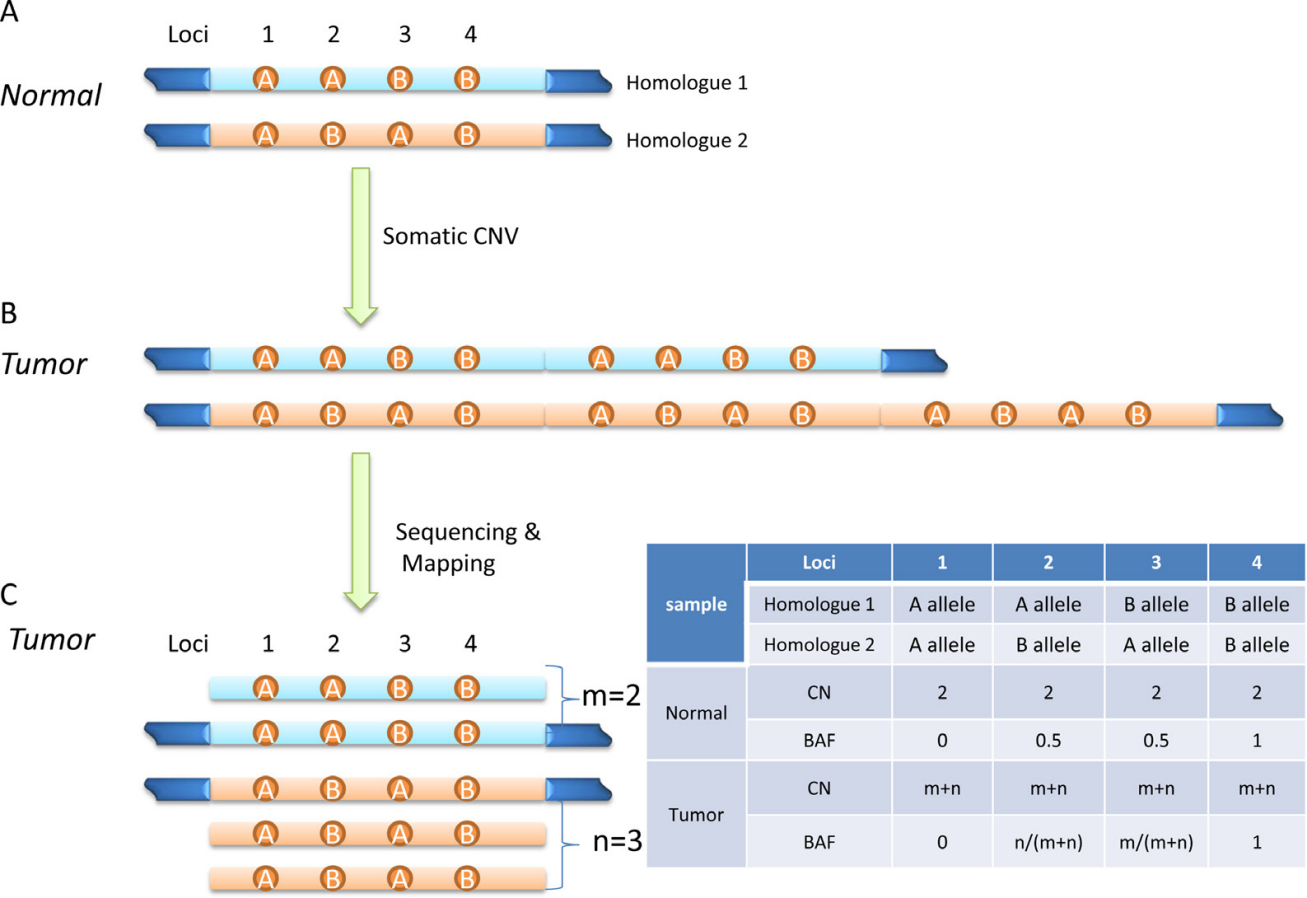
Diagram of detecting somatic CNV from sequencing data (A) A normal human genome usually has two copies of its chromosomes (each copy or homologue from either parents), and contains loci with different genotypes (AA, AB, BA, and BB for loci 1-4, respectively). (B) A somatic CNV event (tandem duplication here) alters copy number of some genomic regions. (C) Pileup view of mapped reads. Altered relative read depth or read counts can be observed. Depending on copy numbers of two homologues in tumor genome, shifted B allele frequency might be observed at heterozygous loci (see the table. CN, copy number; BAF, B allele frequency).

A normal human cell usually has two copies of its genetic materials (Homologues 1 and 2), with one copy from each parent (Figure 2A). When a CNV event happens in a genomic region, it becomes aneuploid, *i.e.* its copy number deviates from 2. The event could be a deletion (loss of genetic material), or amplification (gain of genetic material). Figure 2B shows a simple case with tandem amplification. NGS technology is capable of producing short reads of 100-150 bases in length, which will be mapped to the reference genome. A basic hypothesis in NGS is that each read is a random representation of the targeted regions or the whole genome, thus the mean or median read depth (RD) or read counts (RC) of a genomic region should be proportional to its abundance, or say, its copy number (Figure 2C). Here, read depth (RD) is defined as the number of reads covering a specific locus in the alignment file; read count (RC) is the number of reads falling into a region in the reference genome. RD and RC are two different ways to describe the frequency of a genomic unit (base pair or segment) being represented in sequencing data. They are usually represented in the log2 scale and relative to a selected reference value, and called Log RD Ratio or Log RC Ratio (LRR). LRR is the primary information used in most NGS-based analytic tools for extracting copy number. In principle, LRR provides enough information for CNV detection. However, inherited data bias, intrinsic sample characteristics, and random experimental variations make it problematic to call CNV solely on LRR. Other information, especially B Allele Frequency or Fraction (BAF), could aid CNV detection.

### BAF

As illustrated in Figure 2A, BAF is the estimation of allelic fraction at a SNP locus. Alleles are assigned arbitrarily, but usually the allele with the same nucleotide as the reference genome is assigned as ‘A’ allele, and the allele with different nucleotide from reference is called ‘B’ allele. BAF is calculated as b/(a+b) at each SNP locus, where a and b are copy number of A and B allele, respectively. There are four genotype possibilities for a SNP locus in a normal genome: AA, AB, BA, and BB, and their BAFs are 0, 0.5, 0.5, and 1, respectively (Figure 2). Genotypes AB and BA are not distinguishable from their BAFs. When the copy number is away from 2, BAF has other possibilities, which depend on allelic copy number. As illustrated in Figure 2c, if there are *m* copies of Homologue 1 and *n* copies of Homologue 2 in a tumor genomic region, BAF has possibilities of 0, *m/(m+n), n/(m+n)*, and 1 (table in Figure 2). Comparing tumor and normal genome, the BAF at heterozygous loci (Loci 2 and 3 in Figure 2) may shift away from 0.5 in tumor genome. A CNV segment usually contains many heterozygous SNP loci, so the BAF plot of heterozygous SNP loci across the tumor genome segment may split into two bands at mean value of *m/(m+n)* and *n/(m+n)*, respectively, while it is only one band at mean value of 0.5 across the normal genome segment. Presumably, BAF is more sensitive to CNV events than LRR, since biases introduced by local nucleotide sequence (for example, GC bias) are canceled in BAF calculation. While BAF shifting away from mean value of 0.5 indicates a CNV event in the corresponding genomic region, BAF alone does not provide enough information to identify absolute tumor copy number especially when normal tissue contamination exists. Moreover, false negative calls are possible if CNV calling is solely based on BAF, since BAF remaining at mean value of 0.5 does not indicate lack of CNV events in the region. In the scenario of homozygous amplification, *m* equals *n* so that the BAF possibilities are the same as normal diploid genome, and LRR is necessary for determining copy number. Therefore, accurate CNV detection will likely benefit from the combination of LRR and BAF.

As show in Table 2, all existing programs exploit the LRR (either RD or RC), and several have incorporated BAF information. Patchwork incorporates LRR and BAF for pattern recognition, as only certain LRR and BAF combinations are possible. HAPSEG/ABSOLUTE has the option of matching pattern of local relative DNA concentration to interpret the segments. The terms of local relative DNA concentration is equivalent to the combination of total copy number and BAF in a segment, since relative DNA concentration could be derived from total copy number and BAF, and *vice versa*. CONSERTING has the option to use regions with BAF equals 0.5 for determining diploid baseline.

### Soft-clipped reads and discordant read pairs

New nucleotide sequences often arise in the boundary regions when a CNV event occurs (Figure 2B). These new sequences might be captured by soft-clipped reads or discordant read pairs in WGS data, and the probability of them being captured by WES methodologies is low. Soft-clipped reads are reads whose sequences are mapped to discrete regions in the reference genome; discordant read pairs are read pairs whose spans and/or orientations are inconsistent with the reference genome. Though it is unlikely to determine segmental copy number solely based on soft-clipped reads and/or discordant read pairs, they could provide useful information to confirm CNV events and to refine the breakpoints. Soft-clipped reads have been combined with LRR and BAF in CONSERTING to detect somatic CNVs.

In short, NGS provides multidimensional data at base level resolution to reveal somatic CNVs. An accurate somatic CNV prediction could be supported by evidences at different levels, including LRR, BAF, soft-clipped reads, discordant read pairs, or their combination.

## Data Preprocessing

The principle in most CNV detection programs is that larger or smaller than expected LRR in a genomic region reflects gain or loss of DNA in this region, respectively. However, random variations and systematic biases including mappability bias and GC-content bias (see below) deviates LRR from “correct” number. It is important to correct the biases and create a baseline for capturing the technical variation of a platform. Then a hypothesis of data (LRR and/or BAF) distribution is needed for segmentation.

### Matched normal

A typical strategy to cancel biases and variations in NGS-based cancer CNV studies is to use sequencing data from matched normal tissue or germline of the same patient (most programs) or another individual with majorly diploid genome (Patchwork) under the identical experimental conditions. This is required or preferred for all somatic CNV detection algorithms listed in Table 2, except ReadDepth, which use only tumor NGS data for CNV detection. Though it is tempting to design an algorithm to detect somatic CNV from tumor sample alone, Xi *et al* oppose this conduction as they believe this would result in many false positive due to the fluctuation of reads distribution along the genome [58]. Moreover, it is important to have matched normal from same patient in somatic CNV detection, because the matched normal helps to identify heterozygous SNP loci for calculating BAF and to filter out benign CNV in patient. While the inclusion of match normal is a powerful strategy for somatic CNV detection, it might not cancel all the biases, and further corrections for mappability and GC-content biases are proposed in several programs.

### Mappability bias

NGS generates short reads in length of 100-150 bases, which are mapped to a reference genome for downstream analysis. The great advantage of using short reads is that it can reach massively parallel sequencing with reasonably low error rate (< 0.1%). However, it brings challenges in mapping, as some reads cannot be uniquely mapped to the reference genome, which are called multi-reads. For a given genome, the ratio of multi-reads in all reads from a platform depends mostly on the length of sequence reads, the number of mismatches allowed in mapping [67], and sequencing approach (pair-end *vs.* single-end sequencing). Mappability is defined as the probability for a region in the reference genome that a read originating from it is unambiguously mapped back to it. It can be calculated by programs such as GEM mappability [68]. Regions with higher mappability have more unique sequences and produce less ambiguous reads, and *vice versa*. Mutations and/or sequencing errors in just one or two positions in low mappability regions may cause the reads to be mapped to wrong position. This is especially common for repetitive regions. Different strategies are used for dealing with multi-reads: (1) discarding the reads; (2) choosing a random position out of all of equally good match position; (3) reporting all possible positions. No matter what strategy is used, the ambiguous reads will likely create some biases in the read depth or read counts and might cause errors in CNV detection [69]. In theory, discarding multi-reads may lead to false positive deletion calls, while placing a multi-read at a random possible position may cause false negative deletion/amplification calls. The list of programs implementing mappability correction includes ReadDepth, Control-FREEC, HMMCOPY and CONSERTING. Control-FREEC and CONSERTING skip the regions with low mappability (default < 0.85 and 0.9 in Control-FREEC and CONSERTING respectively), and only reads falling in high mappability regions are used to call CNVs. HMMCOPY and OncoSNP-SEQ correct mappability bias in read counts by dividing the raw read counts by regional mappability (Appendix, Equation 1). In this way, ambiguous reads will be discarded, and unambiguous reads in low mappability regions have bigger weight for CNV detection than reads in high mappability region. To prevent overcorrection, ReadDepth uses the same formula to correct RD data in only high mappability region (default >0.75) and ignores the RD data in low mappability region.

### GC-content bias

It is well known that average read depth of a bin or read count in a region has a unimodal relationship with its GC content, regardless of the chosen bin/region size or average coverage [70-72]. Bins with high or low GC-content have lower mean read depth than bins with medium GC-content (40% to 55% GC). This is believed partially due to PCR efficiency in amplification [71] and sequencing. What makes correcting the bias even harder is while read depth *v.s.* GC-content curves are all unimodal, different samples or even repeated experiments have different slopes, locations of modes, and variances [71]. Benjamini and Speed developed a correction method based on the fragment and fragment-length models to remove most GC-dependent fragment count variation [71]. The method has been implemented in HMMCOPY program, which is based on read counts. When it is applied to read depth but not read counts, increased overall read depth variance was observed [69]. In currently available read depth based programs including CONSERTING, ReadDepth, and Patchwork, the GC bias is corrected in a fashion described by Teo *et al* [69] and Yoon *et al* [72] (Appendix, Equation 2). Regions with extreme GC content (high or low) might be excluded from the analysis. Control-FREEC and OncoSNP-SEQ uses this way for correcting GC bias too, though they are read counts based methods.

### Other biases

Besides mappability and GC-content, there might be additional biases in NGS data which haven't been explicitly corrected in existing CNV detection programs. For example, A and T are more common near the fragment ends, and fragments are much more likely to start with a CpG dinucleotide than any other dinucleotides when the fragment libraries are prepared following Illumina procedure [71]. These local biases near fragment ends might imply that the fragmentation is not truly random. Moreover, it is not clear how Phred-score filtering of sequence reads affect CNV detection [69]. Further investigations are necessary for systematical bias correction in NGS-based cancer CNV studies [73-76].

### Assumption of data distribution

Supposing all the biases are removed from NGS data, an assumption of data distribution is needed to model the data variation for segmentation in most CNV detection programs (except BIC-seq which uses Bayesian Information Criterion (BIC) as merging and stopping criterion). Since the sequence reads are assumed to be chosen randomly from the genome, the RC or RD in a region should follow a Poisson distribution with mean directly proportional to the size of the region and to the copy number [41]. In most of the programs, the hypothetical Poisson distribution is approximated to normal distribution. However, Miller *et. al.* found that the observed distribution violates the Poisson distribution's assumption of equal mean and variance, and negative-binomial distribution is a better approximation for the over dispersed Poisson distribution [57]. As a result, negative binomial distribution is used for bias corrected NGS data in the ReadDepth program. In OncoSNP-SEQ, a mixture of uniform and binomial distribution is used. Overall, an improved understanding of NGS data distribution will likely improve the detection accuracy.

## Segmentation

Segmentation is the process that combines all the reads from same continuous region into a segment with determined boundaries. The challenge in segmentation is that the algorithm needs to distinguish the data variation caused by genuine CNV from that by random effects. Several strategies have been used for this purpose. Two of the most widely used segmentation modules in CNV detection algorithms for array CGH and SNP array, Circular Binary Segmentation (CBS) [77-79] and Hidden Markov Model (HMM) [80-82], have been adapted into programs for NGS data. CBS is used in Patchwork, ExomeCNV, and VarScan2; HMM is implemented into OncoSNP-SEQ and HMMCOPY. The key idea of CBS is joining the ends of a chromosome to make a circle and then iteratively computing segments to minimize the variance within segments and maximize the variance between segments. HMM simultaneously classify each window into a fixed number of possible states based on the read count in the window via an emission distribution (usually Guassian), and make segmentation by combining consecutive windows with same states. Under HMM, segmentation and classification can promote each other by allowing probabilistic parameters in model efficiently learnt from data through algorithms like Expectation Maximization (EM). However, OncoSNP-SEQ uses fixed parameters obtained by off-line training in the model, because the iterative application of the forward-backward algorithm for HMM is not computational trivial due to the sequence length and dynamic range. Besides CBS and HMM, some new algorithms have been developed based on NGS data in the past several years: Xi *et al* developed the BIC-seq algorithm for read depth segmentation via minimizing Bayesian Information Criterion (BIC) by merging the appropriate neighboring bins [58]; Chen *et al* designed a regression tree algorithm based on WGS data to integrate read depth change with structure variation sequence signature to determine segments in CONSERTING; Carter *et al* built a probabilistic method by partitioning the genome into segments of distinct copy number and modeling the four distinct genotypes into each segment in HAPSEG/ABSOLUTE [63, 64]; Chiang *et al* used a local change-point analysis with a subsequent merging procedure in SegSeq [41]; Boeva *et al* adapted Lasso algorithm in Control-FREEC to catch the change points [66]. These segmentation algorithms offered a good amount of choices for segmentation, and a systematic evaluation of them using NGS data will be valuable to help researcher choose an appropriate one for their research projects.

## Segments Interpretation

An ideal segmentation approach will merge adjacent data points with same copy number into one segment and divide regions with different copy numbers into different segments. Further step of interpretation is needed to determine the copy number state of each segment, except for HMM based programs (*i.e.*, HMMcopy), which simultaneously classify each data point to a state and merges the points to segments through EM algorithm. To assign a copy number state to each segment, quantitative criteria are necessary. As shown in Table 2, most of the currently available programs interpret the copy number based on specified LRR cutoffs. Some of the programs, including CONSERTING, Control-FREEC, BIC-seq, and VarScan2, set empirical cutoff values to define copy number states. Some other programs, including ExomeCNV, SegSeq, and ReadDepth, optimize the cutoffs to reach the desired sensitivity and specificity. If a segment does not have sufficient coverage to achieve the desired sensitivity/specificity, no call will usually make in order to prevent false call from inferior data quality. The LRR *versus* BAF pattern has been used in Patchwork for assigning copy number state. As LRR *versus* BAF pattern involves two data types, cutoff values of LRR and BAF corresponding to different copy number states are needed. In Patchwork, the empirical cutoff values will be specified by the users. HAPSEG/ABSOLUTE fits observed data to a platform-dependent error model to determine the cutoffs.

## Challenges in Somatic CNV Detection

In theory, ‘digital karyotyping’ is simple and powerful to asses CNV in designated regions from WGS or WES data [83-85]. However, accurate determination of somatic CNVs is still a great challenge, largely due to the complexities of tumor samples. First, CNVs are very extensive and diverse in tumor genome. Second, tumor samples are inevitably contaminated by normal tissues without known fractions. Third, the ploidies of tumor cells are usually unknown. Fourth, multiple clones in tumor sample are possible, owing to subclonal evolution. These issues are further confounded by signal variation caused by local sequence content and by sample quality and experiment conditions, which are proven to be hard to deconvolute in germline samples [86].

### Extensive and diverse CNV events in tumor genome

Germline and somatic CNVs are very different in their extensities and diversities in genome. Overall, germline CNVs covers about 3.7% [35] to 12% [87] of the genome and they often overlap in genomes of different people, while somatic CNVs could compass the whole genome and recurrent ones are at relative low rate. It is usually assumed that non-recurrent and sharp read depth changes are due to technical variations in normal genome sequencing data, but this assumption could be fallacious for tumor samples. These features make some excellent germline CNVs detection programs, such as ERDS [34], JointSLM [88], and CoNIFER [89], not suitable for somatic CNV detection. For example, modeling across samples may improve the performance of germline CNV detection by removing non-recurrent signals variation [88, 90], but this strategy may increase false negative rate in somatic CNV detection because non-recurrent ones are more common in tumor genome.

### Tumor purity

Normal cell contamination in tumor sample will diminish the observed LRR changes caused by CNVs, and shift BAFs away from presumed values. This introduces difficulties in determining segmental copy number based on LRR and BAF, as the cutoff values will depend on tumor purity which is usually unknown. As overall RD or RC of a sample is the linear combination of RD or RC of tumor and normal genomes, fitting measured LRR and/or BAF values across the genome to different tumor percentage could help determine the most likely tumor purity.

### Tumor ploidy

Aneuploidy of tumor genome creates difficulties in determining the copy number state of LRR baseline. NGS experimental protocol constrains the amount of DNA, not the number of cells. Therefore, instead of corresponding to diploidy as in normal human cells, the LRR baseline is corresponding to the average ploidy, which is usually unknown in tumor sample. Combining LRR and BAF information might be able to reveal the average ploidy, since different ploidy has different possibilities of BAF pattern. For example, diploidy has BAF possibilities of 0, 0.5, and 1; tetraploidy has BAF possibilities of 0, 0.25, 0.5, 0.75, and 1. Since tumor purity and ploidy confound with each other, solving them coordinately will likely provide important information to make accurate CNV calls. Patchwork, OncoSNP-SEQ and ABSOLUTE provide function to evaluate tumor purity and ploidy. Control-FREEC asks users to input sample ploidy, and it can estimate tumor purity. If ploidy is not known, it is suggested to run the program several times with possible ploidy values and compare the results.

### Tumor heterogeneity

Multiple clones of tumor cells could coexist in one tumor [91], and subclones are important to tumor evolution and cancer relapse. Due to their low percentage in a sample, it is hard to determine the subclones. While increasing the depth of sequencing can help to capture the substantial subclone, the accuracy also depends on the properties of tumor sample and the complexity of tumor genomic being investigated. OncoSNP-SEQ and ABSOLUTE provide the feature to detect heterogeneous events.

For a given copy number state, only certain LRR and BAF combinations are possible. Therefore, the number of LRR vs BAF patterns is limited. The deviations from these patterns are usually caused by tumor impurity, aneuploidy, tumor heterogeneity, or their combinations. The observed LRR and BAF of a tumor sample are the linear combinations of LRR and BAF of its components [64], when there are admixtures of normal contamination and multiple clones of tumor cells. From the deviation of patterns, it is possible to deconvolute the tumor impurity, aneuploidy, and tumor heterogeneity.

### Lack of gold standard

Another challenge in somatic CNV detection algorithm development is the lack of gold standard controls or samples to benchmark CNV calling results. Though all the published NGS based somatic CNV detection programs have been tested by some sort of benchmark, such as *in silico* simulated data or calls from SNP arrays on the same set of tumor samples, the generality of such benchmark is less clear. While several simulators, such as ART [92], pIRS [93], GemSIM [94], and Wessim [95], have been developed to simulate NGS data by reproducing known biases from sequence context and empirical platform-dependent error, there is no comprehensive tumor genome simulator that captures all the features of tumor genome mentioned above. Meanwhile, it has been shown that there is a striking lack of reproducibility and concordance for array-based platforms and calling algorithms based on recent assessment [86]. Without a well controlled reference set, it will be difficult to further understand the advantages and disadvantages associated with each program. As a result, the choice of NGS based CNV detection algorithm relies more on factors such as the algorithm's technique descriptions instead of its performances with a common benchmark. Therefore, it is necessary for the community to establish better benchmark datasets, which compass the complexity of tumor genome, for algorithms evaluation and further development.

## CONCLUSIONS AND OUTLOOKS

One of the most important somatic aberrations, CNV in tumor genomes is believed to have high probability of harboring oncotargets. The widespread availability of NGS technology provides an unprecedented opportunity to systematically screen for somatic CNVs. Accurate detection of somatic CNV from massive amount of raw sequence data for each individual requires sophisticated computational algorithms. Read depth or read counts, BAF, soft clipped reads, and discordant reads pairs derived from sequence read mapping are the primary input for CNV determination. During the past several years, a number of computational algorithms have been developed to retrieve copy number from one or more of these data types. In this article, we reviewed 11 existing programs for determining somatic CNV from NGS data, described their similarity and difference in types of data using, data preprocessing, data segmentation, and data interpretation, and highlighted challenges associated with the analysis of NGS data for CNV detection in cancer studies Our review serves as a timely and practical guide to the analytic tools used in NGS-based cancer CNV studies.

Due to the special characteristics of tumor samples and the extraordinary complexity of tumor genomes, accurate detection of somatic CNVs is still a great challenge for the community. Improved computational frameworks are required to take full use of NGS data in order to tackle the tumor purity, ploidy, and heterogeneity coordinately for allele specific copy number calling. Meanwhile, standard protocols, quality control measures, and benchmark are much needed for better further understanding of the advantages and disadvantages associated with existing programs, and to foster the development of next-generation analytic tools.

## Supplementary Appendix


