# Supplementary material for: Computational methods for detecting copy number variations in cancer genome using next generation sequencing: principles and challenges
Source: Oncotarget. 2013 Nov 16;4(11):1868–81. doi: 10.18632/oncotarget.1537 (PMC3875755; doi:10.18632/oncotarget.1537)
Supplement: Supplementary file 1 [file oncotarget-04-1868-s001.doc]

**Appendix: Computational Methods for Detecting Copy Number Variations in Cancer Genome using Next Generation Sequencing: Principles and Challenges**

**Equation 1:**


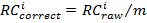


Where index i is the bin index, *RC_correct_* is the read counts after correction, *RC_raw_* is the read counts before correction, and *m* is regional mappability after locally weighted scatter plot smooth.

**Equation 2:**


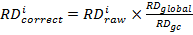


Where
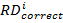
 is the corrected read depth in bin
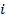
;
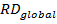
is median read depth of the chromosome, and
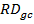
 is median read depth in windows with same GC content in the chromosome.
